# Supplementary material for: Bioinformatic analysis and identification of potential prognostic microRNAs and mRNAs in thyroid cancer
Source: PeerJ. 2018 May 4;6:e4674. doi: 10.7717/peerj.4674 (PMC5937477; doi:10.7717/peerj.4674)
Supplement: Table S1 [file peerj-06-4674-s001.docx]

Table S1. Summary of analysis of papillary thyroid cancer

| **Study** | **Data type** | **GSE ID** | **Sample count (case:control)** |
| --- | --- | --- | --- |
| Ting Qu, 2016 | gene expression arrays | GSE3467  GSE3678 | 16:16 |
| Xinyong Zhu, 2014 | gene expression arrays | GSE3678 | 7:7 |
| Zhao M, 2016 | gene expression arrays | GSE53157 | 7:3 |
| Wei Zhu, 2013 | gene expression arrays | GSE3467 | 9:9 |
| Minna E, 2016 | miRNA expression arrays | GSE73182 | 19:5 |
