# Supplementary material for: Bioinformatic analysis and identification of potential prognostic microRNAs and mRNAs in thyroid cancer
Source: PeerJ. 2018 May 4;6:e4674. doi: 10.7717/peerj.4674 (PMC5937477; doi:10.7717/peerj.4674)
Supplement: Table S2 [file peerj-06-4674-s002.docx]

Table S2. miRNAs in papillary thyroid cancer

| **Study** | **miRNAs** |
| --- | --- |
| He H, 2005 | miR-21, miR-146, miR-181, miR-221, miR-222 |
| Cahill S, 2006 | miR-15a, miR-34a, miR-34c, miR-96, miR-99a, miR-100, miR-107, miR-125b, miR-127, miR-128b, miR-130b, miR-135b, miR-139, miR-141, miR-142-3p, miR-145, miR-146, miR-148, miR-149, miR-154, miR-181a, miR-185, miR-200a, miR-200b, miR-211, miR-213, miR-216, let7d, miR-218, miR-299, miR-302b, miR-302c, miR-323 and miR-370 |
| Pallante P, 2006 | miR-181b, miR213, miR-220, miR-221 and miR-222 |
| Tetzlaff MT, 2007 | miR-19b-1,2, miR-21, miR-30a-5p, miR-30c miR-31, miR-34a, miR-130b, miR-145, miR-172, miR-181a, miR-181b, miR-213, miR-218, miR-221, miR-222, miR-223, mir-224, miR-292-as, miR-300 and miR-345 |
| Chen YT, 2008 | miR-146b, miR-221 and miR-222 |
| Visone R, 2007 | miR-221 and miR-222 |
| Chou CK, 2010 | miR-146b, miR-221 and miR-222 |
