# Supplementary figures and images for: Bioinformatic analysis and identification of potential prognostic microRNAs and mRNAs in thyroid cancer
Source: PeerJ. 2018 May 4;6:e4674. doi: 10.7717/peerj.4674 (PMC5937477; doi:10.7717/peerj.4674)

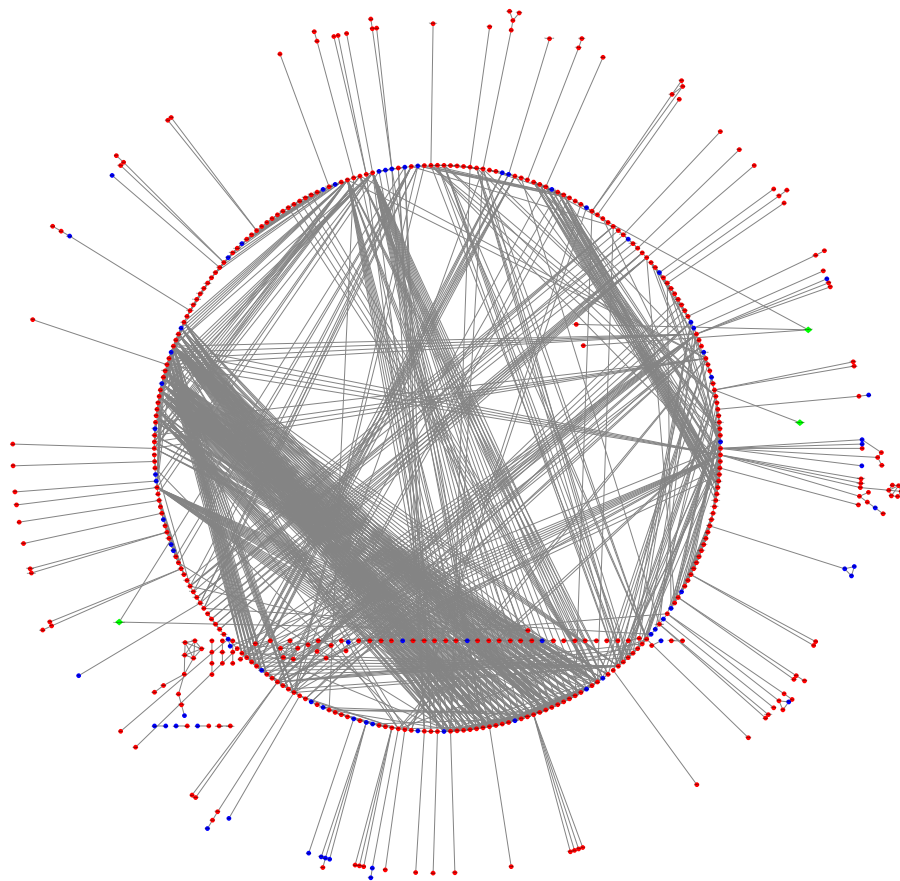

Supplement: Figure S1 — The red nodes represent up-regulated mRNAs, blue nodes represent down-regulated mRNAs and green nodes represent microRNAs. [file peerj-06-4674-s003.pdf]

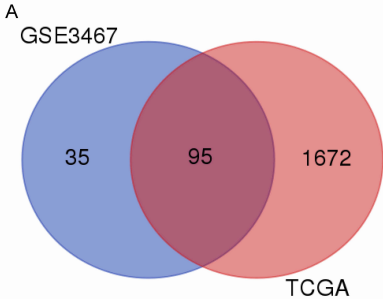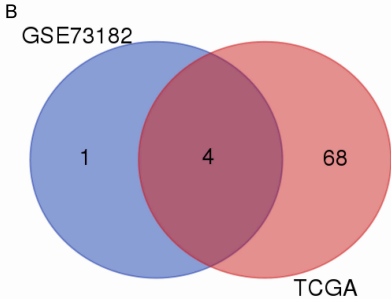

Supplement: Figure S2 — (A) overlapping differentially expressed mRNAs. (B) overlapping differentially expressed miRNAs. [file peerj-06-4674-s004.pdf]
